# Micro-Leakage Image Recognition Method for Internal Detection in Small, Buried Gas Pipelines

**DOI:** 10.3390/s23083956

**Published:** 2023-04-13

**Authors:** Yuxin Zhao, Zhong Su, Hui Zhao

**Affiliations:** 1 School of Automation, Beijing Information Science & Technology University, Beijing 100192, China; 2 Beijing Key Laboratory of High Dynamic Navigation Technology, Beijing 100192, China

**Keywords:** image sample diversity, internal detection, microleakage image recognition, small buried gas pipeline, small target detection layer

## Abstract

In order to resolve the problem that the sample of image for internal detection of DN100 buried gas pipeline microleakage is single and difficult to identify, a recognition method of microleakage image of the pipeline internal detection robot is proposed. First, nongenerative data augmentation is used to expand the microleakage images of gas pipelines. Secondly, a generative data augmentation network, Deep Convolutional Wasserstein Generative Adversarial Networks (DCWGANs), is designed to generate microleakage images with different features for detection in the pipeline of gas pipelines to achieve sample diversity of microleakage images of gas pipelines. Then, a bi-directional feature pyramid network (BiFPN) is introduced into You Only Look Once (YOLOv5) to retain more deep feature information by adding cross-scale connecting lines in the feature fusion structure; finally, a small target detection layer is constructed in YOLOv5 so that more shallow feature information can be retained to achieve small-scale leak point recognition. The experimental results show that the precision of this method for microleak identification is 95.04%, the recall rate is 94.86%, the mAP value is 96.31%, and the minimum size of identifiable leaks is 1 mm.

## 1. Introduction

With increased reliance on gas, higher demands are being placed on the reliability and safety of pipelines, which are the primary carriers of gas [1,2,3]. At present, the commonly used energy transmission methods mainly include highway, waterway, railroad, aviation, pipeline, etc. Among them, pipeline transmission is widely used in gas transmission for its advantages of large transmission capacity, economy, safety, stability, convenience and efficiency [4,5], etc. It has become the main carrier of gas transmission and brought great economic benefits to industrial development and people’s lives. Most gas pipelines are buried underground. Due to the different times and ways of burial, the pipelines are getting longer and more complex, and most of them are in the late design life leading to inevitable problems during the use of the pipelines [6,7]. Pipeline aging, wear, corrosion, and external forces may lead to leak points [8,9,10,11], which not only reduces the efficiency of pipeline transport, but also causes economic losses and environmental pollution [12,13]. Natural gas has a low explosion limit, and the leaking gas is flammable and explosive when it reaches a certain concentration, which can easily cause major safety accidents that seriously threaten the lives and properties of the general public [14,15]. The current mainstream gas pipeline leak identification method is outside the pipeline detection, but outside the pipeline, detection excavation cost is high and can only identify the size of the larger leak points [16,17,18,19] and cannot meet the requirement of 1 mm minimum identifiable leak size for DN100 buried gas pipeline. Therefore, the design of a microleakage image recognition method applicable to the detection inside small, buried gas pipelines is of great significance to ensure people’s safety and enhance economic efficiency.

**Figure 1:**
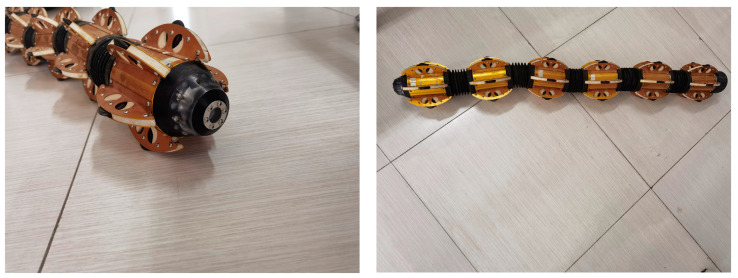
In-pipe detection robot.

**Figure 2:**
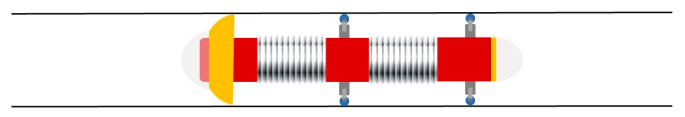
Working diagram of the internal pipeline inspection robot.

**Figure 3:**
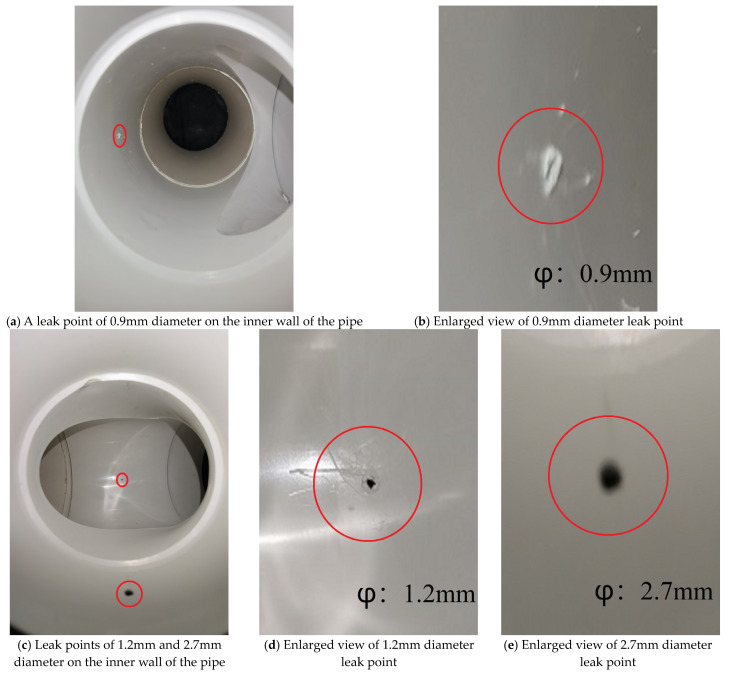
DN100 gas pipeline and its leakage point local enlargement.

The proposed leak identification method for internal pipeline inspection of small, buried gas pipelines is based on an internal pipeline inspection robot. The internal pipeline inspection robot uses a multi-section structure, with each two sections bendable at a certain angle, and a vision sensor installed in the head for collecting images of micro-leaks in the pipeline. The internal pipeline inspection robot is shown in Figure 1. The internal pipeline inspection robot can travel inside the DN100 pipeline and can pass through the pipeline paths, such as straight and tee, and take pictures of the inside of the pipe during the travel. The working schematic of the internal pipeline inspection robot is shown in Figure 2. The material of the small, buried gas pipe in this paper is the PE pipe, and the pipe specification is DN100. The leak point of DN100 gas pipe and its local enlargement are shown in Figure 3. Among them, Figure 3a shows a leak point on the inner wall of the DN100 PE pipe with a diameter of 0.9 mm, while Figure 3b shows an enlarged view of this leak point. Figure 3c shows two leakage points of the DN100 PE pipe tee, the diameter of the front leakage point being 1.2 mm. Figure 3d shows the enlarged view of this leakage point, and the diameter of the lower leakage point is 2.7 mm. Figure 3e shows the enlarged view of this leakage point.

For the problem of single sample and difficult recognition of the DN100 buried gas pipeline microleakage internal detection image, we propose a microleakage image recognition method applicable to the internal pipeline inspection robots. The method first expands the number of images by nongenerative data enhancement methods such as rotation, panning, cropping, color, saturation and brightness adjustment, to reduce the risk of overfitting of the leak recognition network. Secondly, the DCWGAN generative data enhancement method was designed based on Deep Convolutional Generative Adversarial Network (DCGAN) [20] with Wasserstein Generative Adversarial Network using Gradient Penalty (WGAN-GP) [21], to generate image samples with different features to achieve image sample diversity and improve the generalizability of leak recognition network. Then, YOLO [22] is used as the target detection algorithm, and the BiFPN structure is introduced into YOLOv5s to retain more deep feature information in feature fusion and improve the network feature fusion capability, recognition precision and recognition efficiency. Finally, a small target detection layer is constructed in YOLOv5s to retain more shallow feature information, improve the recognition of small-scale leakage points, and reduce the rate of missed detection.

## 2. Image Sample Diversity

### 2.1. DCGAN

**Figure 4:**
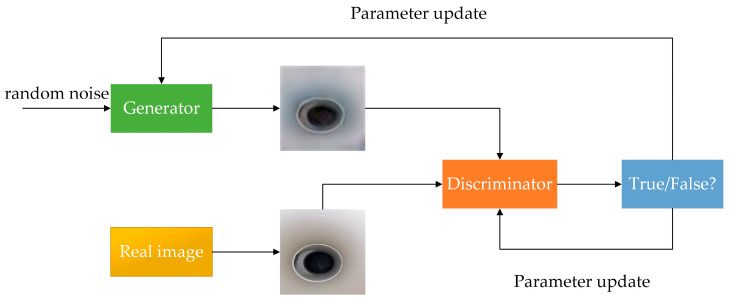
DCGAN structure diagram.

DCGAN consists of a generator and a discriminator, which uses a CNN instead of a multilayer perceptron combined with the original GAN. The generating network continuously optimizes the generated images to make the discriminating network misjudge them, and the discriminating network optimizes itself to make its own judgments more accurate. The DCGAN structure is shown in Figure 4. DCGAN improves stability quality of the generated images by making the following improvements on the basis of GAN:

1. Both the generator and discriminator of DCGAN discard the pooling layer of the CNN, the discriminator retains the overall architecture of the CNN, and the generator replaces the convolutional layer with a deconvolutional layer;
2. Batch normalization layer (BNL) is used in the discriminator and generator to accelerate the model training and improve the training stability. However, since direct application of batch normalization (BN) to all layers leads to sample oscillation and model instability, the batch normalization layer is not used in the output layer of the generator and the input layer of the discriminator;
3. ReLU is used as the activation function in the generator network, and Tanh is used as the activation function in the last layer;
4. LeakyReLU is used as the activation function in the discriminator network to prevent gradient sparsity;
5. Using the Adam optimizer, the value of the exponential decay rate of the first-order moment estimate is set to 0.5.

### 2.2. WGAN-GP

Although DCGAN has improved network performance compared with GAN, the composition of the loss function is still based on the JS scatter, which means that DCGAN still has the problems of slow convergence and easy gradient disappearance during the training process. The loss of function based on the JS scatter is shown in Equation (1):

(1) $$L\left(y_{j},{\hat{y}}_{j}\right)=-\frac{1}{N}\sum_{j=1}^{N}\left[y_{j}\log y_{j}+\left(1-{\hat{y}}_{j}\right)\log_{2}\left(1-{\hat{y}}_{j}\right)\right]$$

The WGAN-GP network uses Wasserstein distance instead of the JS scatter to represent the distance relationship between data distributions. The Wasserstein distance is defined as shown in Equation (2):

(2) $$W\left(P_{1},P_{2}\right)=\underset{ϒ∼\Pi \left(P_{1},P_{2}\right)}{\inf}E_{\left(x,y\right)∼ϒ}\left[∥x-y∥\right],$$

where *x* and *y* are the two samples, *P*_1_ and *P*_2_ are the distributions of the samples, respectively, γ is the joint distribution and inf is the maximum lower bound function. In order to satisfy the Lipschitz continuity condition, this paper uses the Wasserstein distance to form the loss function, a gradient penalty (GP) is introduced in WGAN-GP, and the target loss function is shown in Equation (3):

(3) $$L=\underset{\tilde{x}∼P_{g}}{E}\left[D\left(\tilde{x}\right)\right]-\underset{x∼P_{r}}{E}\left[D\left(x\right)\right]+\lambda \underset{\tilde{x}∼P_{\hat{x}}}{E}\left[{\left(∥\nabla_{\hat{x}}D\left(\hat{x}\right){∥}_{2}-1\right)}^{2}\right],$$

where *x* is the true data, $\hat{x}$ is the generated data, $\hat{x}$ after interpolation of the real data and the generated data where $\hat{x}=\varepsilon x+\left(1-\varepsilon \right)\tilde{x}$.

### 2.3. DCWGAN

**Figure 5:**
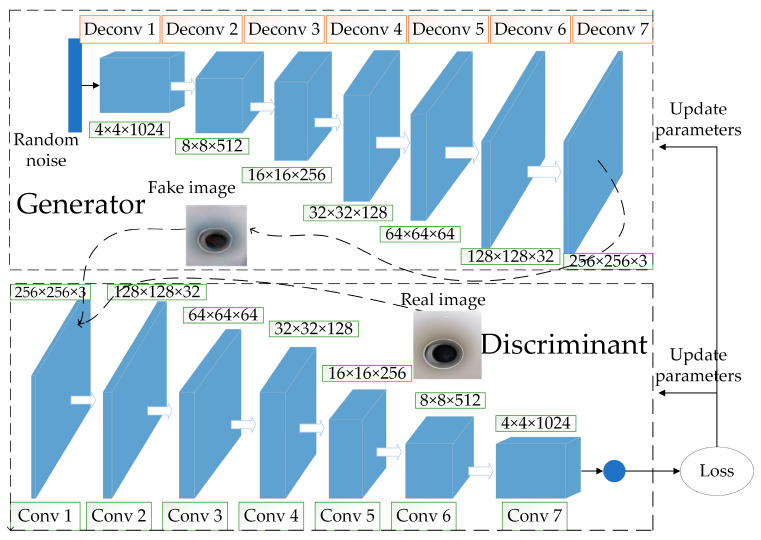
Microleakage generation model for small, buried gas pipelines.

In order to improve the DCGAN training convergence speed and reduce gradient disappearance, we propose the DCWGAN generative data enhancement network. The network is based on DCGAN and combines the advantages of DCGAN and WGAN-GP. Specifically, Wasserstein distance is introduced based on DGGAN instead of the JS scatter to represent the distance relationship between data distributions, and the loss function of WGAN-GP is used instead of the loss function of DCGAN to reduce gradient disappearance, with faster convergence speed and higher quality of the generated images. The microleakage generation model for small, buried gas pipelines constructed by DCWGAN is shown in Figure 5.

#### 2.3.1. Improved Generator

**Figure 6:**
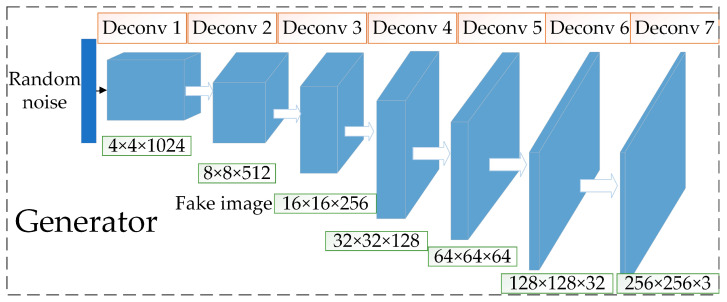
Improved generator.

The improved generator structure is shown in Figure 6, which adds two layers to the five-layer structure of the base network, and contains seven layers of deconvolution layers to improve the quality of the network-generated images. The input is a 128-dimensional random noise vector obeying Gaussian distribution instead of a mean distributed random noise vector, and the output is an image of size 256 × 256 × 3. Unlike the base network, this network uses a fully convolutional network, eliminating the pooling layer and using a deconvolutional layer instead to do the up-sampling operation. The specific implementation steps are: first, a 128-dimensional random noise vector obeying Gaussian distribution is input; secondly, the noise vector is turned into a 4 × 4 × 1024 image using convolution instead of a fully connected layer; then, six deconvolutions are passed in turn, where the number of channels is halved for each deconvolution, and the size becomes twice the original, the final output size is 256 × 256 × 3 for the images; finally, the network adds a layer of BNL after each layer of deconvolution based on the original generator, to prevent gradient disappearance. The ReLU activation function is used for each layer except for the last layer, where the activation function is Tanh.

#### 2.3.2. Improved Discriminator

**Figure 7:**
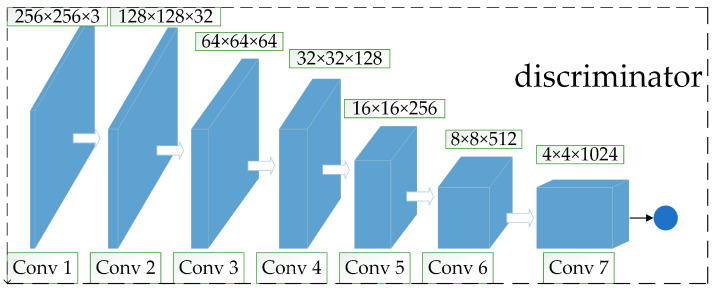
Improved discriminator.

The structure of the improved discriminator is shown in Figure 7. The structure is similar to that of the generator, both are seven-layer structures, the difference being that all seven layers of the discriminator are convolutional layers. The input is a 256 × 256 × 3 image and the output is a one-dimensional prediction used to predict whether the input image is a real image or a generator-generated image. The steps are similar to that of the generator implementation: first, the input is a 256 × 256 × 3 image; next, the final output of the probability value to determine the image is true or false through six layers of convolution, where the number of image channels becomes twice the original and the size is reduced by half after each convolution operation; finally, a BNL layer is also added to each convolutional layer, and the activation function used in each layer is LeakyReLU, except for the last layer, which uses sigmoid.

## 3. Microleakage Image Recognition for Internal Detection

### 3.1. Original YOLOv5s

**Figure 8:**
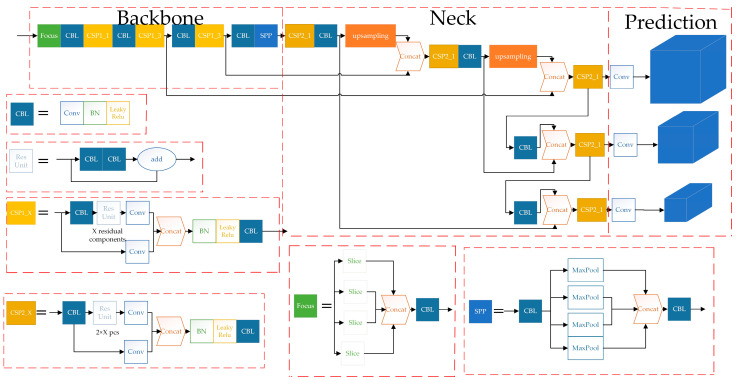
The overall architecture of the original YOLOv5s network.

YOLO is a typical one-stage target detection algorithm that is widely used in target detection tasks. We choose the YOLOv5s network with a small network width and depth as the basic framework for network construction of microleakage image recognition for detection in small, buried gas pipelines, from the perspectives of computing power and storage. The overall architecture of the YOLOv5s [23] network consists of three parts: the backbone, the neck and the prediction, as shown in Figure 8.

The YOLOv5s network has a complex structure and can be made up of a variety of modules. The most basic modules of YOLOv5s include: CBL, Res unit, CSP 1_X, CSP 2_X, Focus, SPP, etc.

The backbone is mainly used to extract features of different scales of the target. Among them, the Focus module slices the input image, doubles the image size and changes the number of feature maps to four times the original size, and then stacks the feature maps to reduce the computation of the network and improve the network speed. The CSP module, referred to as the C3 module, with the help of the idea of CSPNet, divides the feature mapping of the base layer into two parts, and then merges the feature mapping through the cross-stage hierarchy, strengthening the ability of network feature fusion, which can guarantee precision while reducing the amount of computation. The SPP module utilizes a maximum pooling layer of four convolutional kernels of different sizes to effectively increase the range of the backbone features received, significantly separating the most important contextual features and enabling feature fusion at different scales.

**Figure 9:**
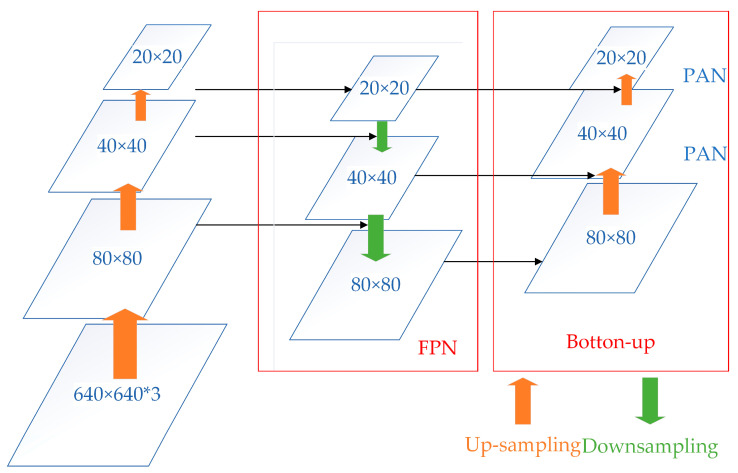
FPN+PAN structure.

The structure of the feature pyramid network (FPN)+path aggregation network (PAN) is used for the neck. Multi-scale feature fusion through sampling operations, FPN+PAN, structure is shown in Figure 9, in which the FPN layer is top–down, and the feature information at the top level is fused by up-sampling to obtain the feature map for prediction. A bottom–up feature pyramid is added behind the FPN layer, including two PAN structures. By combining the above structures, the FPN layer conveys strong semantic features from the top–down, while the feature pyramid behind it conveys strong localization features from the bottom up, and the feature aggregation is performed from a different backbone to different detection layers to further improve the feature extraction capability. 

The three detection heads in Prediction correspond to predicting small, medium and large targets and classifying, as well as predicting the targets, generating target bounding boxes, predicting target categories and confidence information, etc.

### 3.2. MS-YOLOv5s

**Figure 10:**
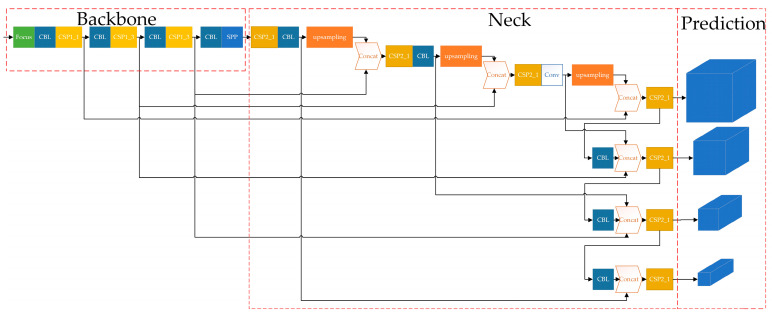
MS-YOLOv5s network architecture.

Although YOLOv5s has a high assessment in target detection, it is still difficult to identify small, buried gas pipeline leak points, especially small targets with a leak size of 1 mm. Therefore, MS-YOLOv5s is proposed in order to enhance the feature expression, improve the precision of the network for leakage points, reduce the leakage detection rate, and improve the detection rate of small leakage points. The MS-YOLOv5s network architecture is shown in Figure 10.

#### 3.2.1. BiFPN

**Figure 11:**
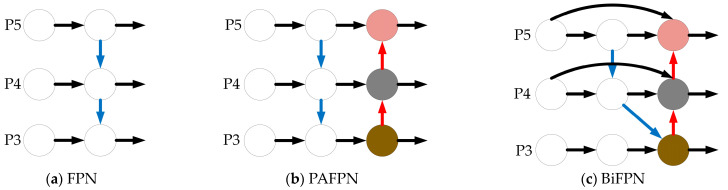
Comparison of different neck network structures.

The comparison of the three different neck network structures, including FPN, PAFPN and BiFPN, are shown in Figure 11. The FPN structure is shown in Figure 11a, which only allows for top–down, unidirectional information flow transfer. The PAFPN structure is shown in Figure 11b, which adds an additional bottom–up path for information enhancement based on the FPN, which preserves more shallow features effectively. We introduce the BiFPN structure in this paper. The cross-scale connection method enables the network to retain more shallow semantic information without losing relatively deep semantic information too much, which reduces the amount of network computation and improves the recognition efficiency, the BiFPN structure is shown in Figure 11c.

FPN, as well as PAFPN structures are based on proximity feature results in the feature fusion process. The BiFPN structure, used in this paper as a feature fusion method, is an efficient bi-directional, cross-scale and weighted feature fusion method to obtain a feature map for multi-scale feature fusion. First, the feature maps from the backbone network at different scales are extracted. Secondly, the first feature fusion is achieved by lateral connection with the down-sampling layer. Then, a second feature fusion with the down-sampled and up-sampled layers at the same scale is achieved by a jump connection. The final feature map is obtained after multi-scale fusion. In the proposed microleakage image recognition method for detection within small, buried gas pipelines, the BiFPN structure as a modular repetitive feature network layer can obtain a more advanced feature fusion method compared to PAFPN, which helps to obtain more shallow features and deep feature fusion information of small, buried gas pipeline leak points, enhance the expression capability of the feature pyramid, improve the leak point recognition precision, recognition efficiency and reduce the leakage detection rate.

#### 3.2.2. Small Target Detection Layer

**Figure 12:**
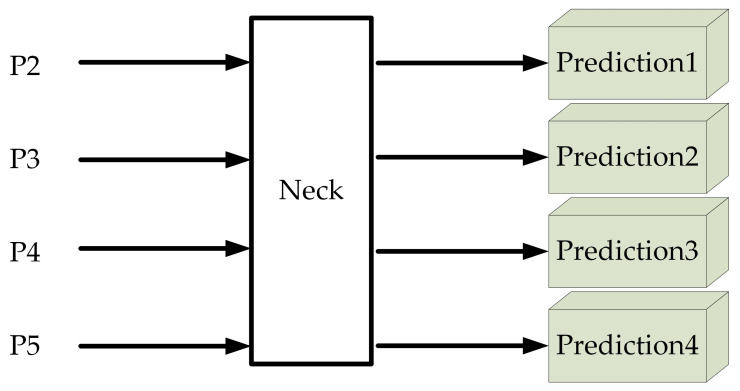
Prediction network optimization.

There are three detection heads in the original YOLOv5s. When the input image is 640 × 640, the detection head output scales are 80 × 80, 40 × 40 and 20 × 20, which are used for small, medium and large target recognition, respectively. However, due to the varying sizes of gas pipeline leak points with a minimum leak size of 1 mm, this paper is improved based on the original YOLOv5s network, in order to improve the detection rate of tiny leak points and reduce the leak detection rate. A new detection head output size of 160 × 160 was added to meet the demand of tiny leak point identification, and the output scales of the final constructed detection layer were 160 × 160, 80 × 80, 40 × 40 and 20 × 20. After constructing the small target detection layer, the output prediction frames are correspondingly increased from 9 to 12, and the three additional prediction frames target the identification of small leaks. The structure of the Prediction network in this paper is shown in Figure 12.

## 4. Experimental Design and Analysis of Results

### 4.1. DCWGAN Network Parameter Setting and Training

The operating system of this experiment is the Win10 hardware platform, CPU is Inter Core i5-13600 and GPU is NVIDIA RTX 3060. The software platforms are Pycharm2022.2.3, Pytorch1.13.0 and Python3.8.

The training parameters are set as shown in Table 1. The training batch size is 64, the generator learning rate is 0.0002, the discriminator learning rate is 0.0002, the number of training iterations is 5000, the decay rate of the optimization index is 0.5, the noise dimension is 128 dimensions and the optimizer is Adam. The generator is first fixed during training. The discriminator takes a portion of the samples from the input image and calculates the loss $\log\left(D\left(x\right)\right)$. Then, a portion of the fake images generated by the generator is acquired and the loss $\log\left(1-D\left(z\right)\right)$ calculated. This process is repeated until the sum of all sample losses is calculated, and the discriminator is updated and optimized using the Adam optimizer. The discriminator is fixed, optimizing generators by generator loss functions $\log\left(1-D\left(G\left(z\right)\right)\right)$.

### 4.2. Image Generation Results and Evaluation

**Figure 13:**
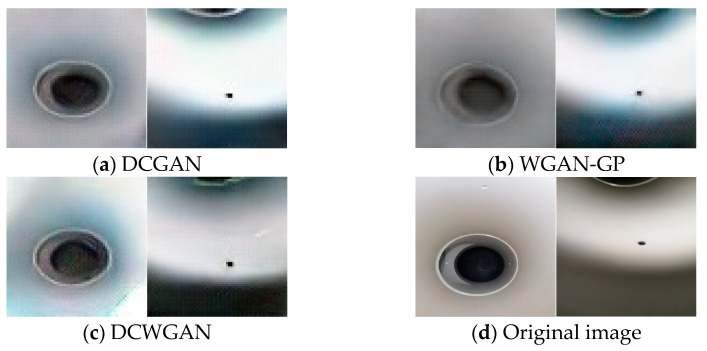
The generated image of each network is compared with the original graph.

Figure 13 shows the comparison of DCGAN-, WGAN-GP- and DCWGAN-generated images with the original images, respectively.

In order to intuitively verify the quality of the DCWGAN-generated images proposed in this paper, the original YOLOv5s algorithm is chosen to verify the DCWGAN-generated images. Average precision (AP) is a composite indicator for the evaluation of identification targets. The comparison of AP values between the original dataset and the dataset with images generated by adding the DCWGAN network verifies that the images generated by DCWGAN can significantly improve the recognition precision of microleakage images of small, buried gas pipelines, which proves the feasibility of the method. The identification results using each dataset are shown in Table 2. The AP value of 67.41% when using the original dataset and AP of 72.73% when using the original dataset+300 generated images can be seen in Table 2. The AP value was 75.15 when using the original dataset+600 generated images. An effectively designed DCWGAN generative data enhancement method achieves image sample diversity and improves the network recognition capability compared to using the original dataset method.

### 4.3. Establishment of Microleakage Image Dataset for Detection in Small Buried Gas Pipelines

The total number of microleakage images of small, buried gas pipelines is 171. Nongenerative data enhancement, including roaming, panning, cropping, color, saturation and brightness adjustments, is performed on the above images to expand the number of data sets without generating new feature information in the process. The DCWGAN generative data enhancement network is designed to achieve the diversity of image samples, improve the generalizability of the recognition network, and further expand the number of datasets.

The dataset built after the nongenerative data enhancement and the generative data enhancement of the DCWGAN designed has a total of 1000 images. The images are annotated using the labeling image annotation tool. The annotated dataset is randomly divided into two groups, of which 80% of the dataset is used for parameter learning and training of the network model, and 20% of the dataset is used to test the recognition ability of the network model.

### 4.4. Experimental Environment

Random initial weights are used, the number of training rounds is 300, the input image size is 640 × 640, the training batch size is 16, the optimizer selects SGD and the number of threads is eight. The network model training parameters are set as shown in Table 3.

### 4.5. Evaluation Metrics

We chose the evaluation indexes commonly used for target detection: Precision (*P*), Recall (*R*), and Multi-class Average Precision (*mAP*). Since there is only one class of gas pipeline leakage points identified as targets, the Average Precision (*AP*) and *mAP* values are equal, and the formulae for calculating *P*, *R*, and *mAP* are shown in Equations (4)–(6), respectively.

(4) $$P_{precision}=\frac{TP}{TP+FP}$$

(5) $$R_{recall}=\frac{TP}{TP+FN}$$

(6) $$mAP=AP=\int_{0}^{1}P\left(r\right)dr,$$

where *TP* is the number of correctly identified leak points, *FP* is the number of leak point misdetections, *FN* is the number of leak point misses, r is the integration variable, which is the integral of the product of precision and recall and *P(r)* denotes the *P*–*R* curve.

### 4.6. Experimental Results

**Figure 14:**
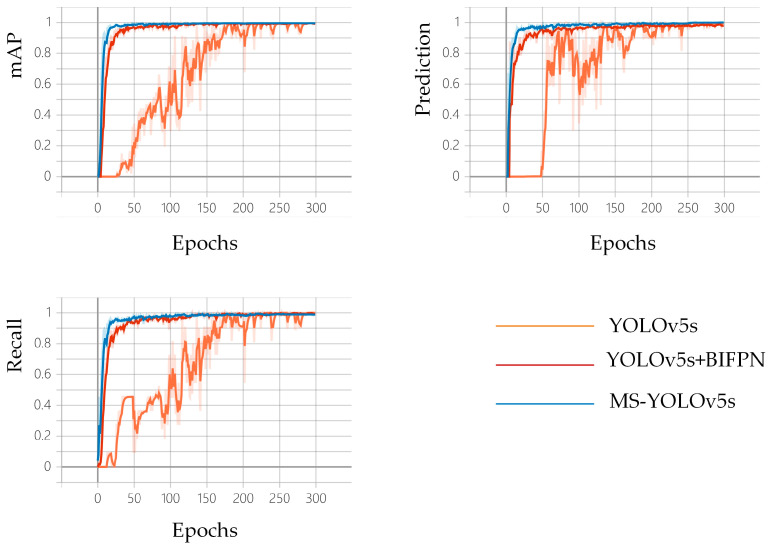
Different model training curves.

The original YOLOv5s networks, YOLOv5s+BiFPN and MS-YOLOv5s, were trained on the same dataset for 300 rounds, respectively. The comparison curves of mAP, Precision, Recall and other metrics of each model are shown in Figure 14. The horizontal coordinates in all three images in Figure 14 are the training rounds. The vertical coordinates indicate the mAP, precision and recall metrics, respectively. From the figure, it can be seen that the original network of YOLOv5s converges around 230 rounds, which is slow to converge. Additionally, the network is less stable and more volatile. The network converges at around 130 rounds after adding the BiFPN structure, with faster convergence and a greater improvement in network fluctuations. The MS-YOLOv5s network converged rapidly at about 70 rounds, the network fluctuations were further improved until the end of training, and the network model worked better without overfitting or underfitting. The training results were better than the original model.

**Figure 15:**
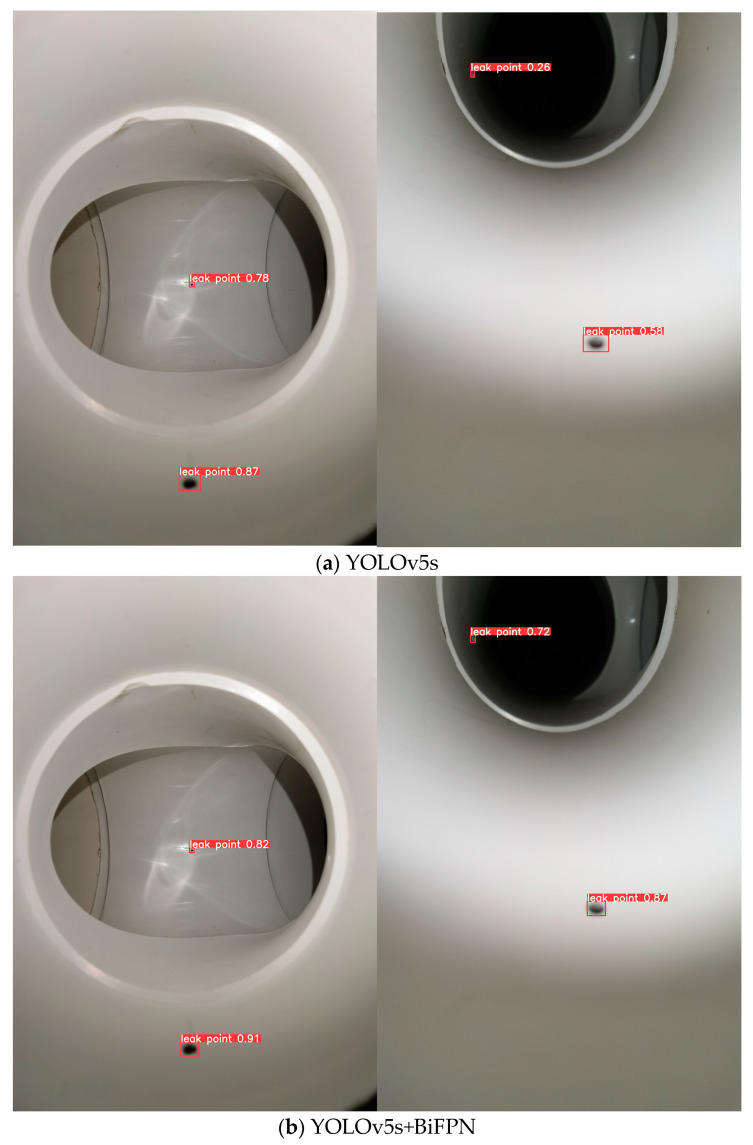

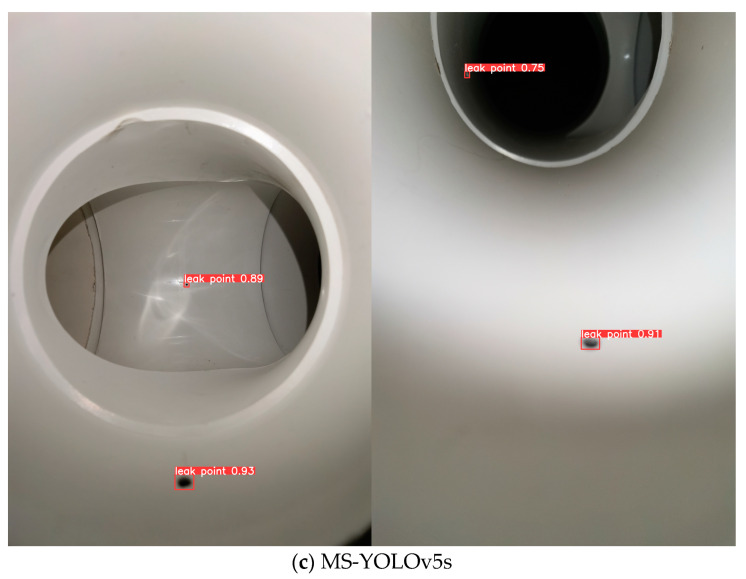
Comparison of recognition results of each network model.

From Table 4, it can be seen that the network, with the addition of the BiFPN structure, has increased the precision by 11.69% to 87.82%, the recall by 7.64% to 84.39% and the mAP value by 10.62% to 88.26% compared to the original network. The MS-YOLOv5s increased precision by 7.22% to 95.04%, the recall by 10.47% to 94.86%, and the average precision by 8.05% to 96.31% compared to adding the BiFPN network. It can be seen that the MS-YOLOv5s network is substantially improved compared with the original network, which can better identify the leakage point, mark the location of the leakage point and give confidence information, the comparison graph of the recognition results of each network model is shown in Figure 15. Figure 15a shows the original YOLOv5s network identification result, Figure 15b shows the YOLOv5s+BiFPN network identification result, and Figure 15c shows the YOLOv5s+BiFPN+P2 network identification result. From these three comparison plots, it can be seen that for the same leak site, the confidence of the YOLOv5s+BiFPN network increases by 4%, 4%, 46% and 29%, respectively, compared to the original YOLOv5s network; and the confidence of the MS-YOLOv5s network increases by 7%, 2%, 3% and 4%, respectively, compared to the YOLOv5s+BiFPN network. Further demonstrating the MS-YOLOv5s network is suitable for microleakage image recognition in small, buried gas pipeline detection.

### 4.7. Discussion

In this study, the number of microleak images detected in small, buried gas pipelines was small, and training YOLOv5s with this dataset would lead to overfitting problems in the network; therefore, a nongenerative data augmentation network was used to expand the number of datasets. However, the expanded image sample is single, and the network model trained on YOLOv5s based on this dataset is not strong in terms of generalization. Therefore, a generative data enhancement network, DCWGAN, is proposed, which combines the advantages of DCGAN and WGAN-GP. The images generated by the DCWGAN network are fed into the YOLOv5s network, and the improvement of AP value proves the feasibility of the DCWGAN network. YOLO is highly evaluated in the field of target detection and has a high recognition capability in most cases, but it often cannot have good results when the recognized targets are small, so improvements are made to YOLO for better recognition capability for small targets, and the feasibility of the proposed MS-YOLOv5s network is demonstrated by comparing it with YOLOv5s+BiFPN and the original YOLOv5s network to achieve better results. However, when we found that the leak point appeared in the background close to the pipe connection and other cases during the actual test, the recognition effect could not achieve satisfactory results. In future works, the images will be pre-processed and the network will be further improved. Thus, the YOLOv5s network may be further enhanced for the recognition of microleakage images for detection in small DN100 buried gas pipelines.

## 5. Conclusions

Aiming at the problem of a single-image sample and difficult recognition of microleakage detected in DN100 small, buried gas pipeline, a DCWGAN generative data enhancement network, based on the nongenerative data enhancement method, is proposed along with a MS-YOLOv5s microleakage recognition method for detection in small, buried gas pipelines.

1. The traditional nongenerative data enhancement method is applied to expand the number of images, the DCWGAN generative data enhancement network is designed to generate images to achieve image sample diversity and further expand the number of images, and a microleakage image dataset for detection in small, buried gas pipelines is established through a combination of the two methods.
2. In this paper, the following steps were taken: designing the MS-YOLOv5s network, introducing the BiFPN structure into the YOLOv5s original network, fusing feature information at different scales, adding cross-scale connecting lines in the feature fusion structure to retain more deep feature information, improving gas pipeline leak point recognition precision and recognition efficiency. The construction of a small target detection layer retains more shallow feature information to enhance the recognition of small leakage points and reduce the leakage detection rate, with the smallest identifiable leak size up to 1 mm. The comparison experiments of different network models show that the Precision, Recall, and mAP values of the MS-YOLOv5s network proposed in this paper are 95.04%, 94.86% and 96.31%, respectively, which have good recognition effects on microleakage points detected in small, buried gas pipelines.

## Figures and Tables

**Table 1:** The parameters of training.

Parameter Name	Value
Batch size	64
Learning rate G	0.0002
Learning rate D	0.0002
Epochs	5000
Betal	0.5
Z	128
Optimizer	Adam

**Table 2:** Identification results.

Training Dataset	AP/%
171 images of pipeline leakage points	67.41
171 images of pipeline leak points + 300 generated images	72.73
171 images of pipeline leak points + 600 generated images	75.15

**Table 3:** Training parameters.

Parameter Name	Value
Weight	Random
Epochs	300
Image size	640 × 640
Batch size	16
Optimizer	SGD
Workers	8

**Table 4:** Experimental results of different models.

Model	Precision/%	Recall/%	mAP/%
YOLOv5s [24]	76.13	76.75	77.64
YOLOv5s+BiFPN [25]	87.82	84.39	88.26
MS-YOLOv5s [26]	95.04	94.86	96.31

## Data Availability

Not applicable.
